# Structural–functional diversity of CD47 proteoforms

**DOI:** 10.3389/fimmu.2024.1329562

**Published:** 2024-02-15

**Authors:** Ting Zhang, Feng Wang, Lu Xu, Yong-Guang Yang

**Affiliations:** ^1^ Key Laboratory of Organ Regeneration and Transplantation of the Ministry of Education, Institute of Immunology, The First Hospital of Jilin University, Changchun, Jilin, China; ^2^ National-Local Joint Engineering Laboratory of Animal Models for Human Disease, The First Hospital of Jilin University, Changchun, China; ^3^ International Center of Future Science, Jilin University, Changchun, Jilin, China

**Keywords:** CD47, alternative splicing, post-translational modification, immunotherapy, CD47-SIRPalfa, immune checkpoints, proteoform

## Abstract

The ubiquitously expressed transmembrane glycoprotein CD47 participates in various important physiological cell functions, including phagocytosis, apoptosis, proliferation, adhesion, and migration, through interactions with its ligands, including the inhibitory receptor signal regulatory protein α (SIRPα), secreted glycoprotein thrombospondin-1 (TSP-1), and integrins. Elevated expression of CD47 is observed in a wide range of cancer cells as a mechanism for evading the immune system, blocking the interaction between the CD47 and SIRPα is the most advanced and promising therapeutic approach currently investigated in multiple clinical trials. The widely held view that a single type of CD47 protein acts through membrane interactions has been challenged by the discovery of a large cohort of CD47 proteins with cell-, tissue-, and temporal-specific expression and functional profiles. These profiles have been derived from a single gene through alternative splicing and post-translational modifications, such as glycosylation, pyroglutamate modification, glycosaminoglycan modification, and proteolytic cleavage and, to some extent, via specific CD47 clustering in aging and tumor cells and the regulation of its subcellular localization by a pre-translational modification, alternative cleavage and polyadenylation (APA). This review explores the origins and molecular properties of CD47 proteoforms and their roles under physiological and pathological conditions, mentioning the new methods to improve the response to the therapeutic inhibition of CD47–SIRPα immune checkpoints, contributing to the understanding of CD47 proteoform diversity and identification of novel clinical targets and immune-related therapeutic candidates.

## 1 Introduction

CD47, also known as integrin-associated protein, was originally discovered as a plasma membrane molecule that copurified with the leukocyte and placental integrin αvβ3. Molecular cloning showed that the protein is identical to the cancer antigen OV-3, the expression of which was higher in ovarian cancer cells than in normal ovarian cells (1–4).

CD47 is a widely expressed five-transmembrane glycoprotein of the immune superfamily that contains an N-terminal extracellular immunoglobulin variable (IgV)-like domain, a multiple membrane-spanning (MMS) domain, and an alternatively spliced short cytoplasmic tail (1, 5). CD47 is best known as a ubiquitous cell surface molecule that acts as a marker of self and protects cells from phagocytosis by macrophages (6) and dendritic cells (7). It plays an important role in mediating cell proliferation, migration, apoptosis, immune homeostasis, inhibition of nitric oxide signal transduction, and other related reactions by binding to secreted glycoprotein thrombospondin-1 (TSP-1), integrins, protein linking integrin-associated protein with cytoskeleton (PLIC)-1/2, and BCL2/adenovirus E1B 19kDa-interacting protein-3 (BNIP3) (8–14). The role of CD47 and their mechanisms have been summarized in several reviews (15–17); however, a systematic overview of the structures and functions of CD47 proteoforms is lacking.

Multiple proteoforms can arise from a single protein-coding gene through genetic variation; alternative splicing of RNA; post-translational modifications (PTMs) to a canonical protein sequence by covalent attachment of chemical functional groups, carbohydrates, lipids, peptides, or proteins; and proteolytic cleavage (18, 19). CD47 is subject to pre-translational modification through alternative splicing of RNA (5) and PTMs, such as glycosylation (2, 20–22), glycosaminoglycan modification (23), pyroglutamate (pGlu) modification (24, 25), and proteolytic cleavage (26–32). Each modification, individually or in combination, generates a conformationally unique CD47 proteoform with varying downstream functionalities and implications.

The extracellular N-terminal IgV domain of CD47 is essential for interacting with the signal regulatory protein α (SIRPα) on the surface of macrophages and dendritic cells, which aids high-CD47-expressing tumor cells and circulating hematopoietic stem cells in avoiding phagocytosis (33–37). Recent clinical trials have focused on blocking the CD47–SIRPα interaction for cancer immunotherapy (34, 38, 39). In addition, transgenic overexpression of CD47 was effective in preventing xenograft and allograft rejection by exerting strong immunoinhibitory effects, providing an effective means to generate hypoimmunogenic pluripotent stem cells, thereby potentially preventing transplant rejection in regenerative medicine (40–42).

However, cell death and inhibition of angiogenesis induced by ligation of CD47 with its ligands, including TSP-1, soluble SIRPα, and agonistic antibodies, are overlooked side effects of CD47 overexpression (32, 43–46). This problem may be overcome by selecting an appropriate isoform or constructing CD47 mutants based on a complete understanding of protein isoform and domain functions. CD47 undergoes N-glycosylation and glycosaminoglycan modification, which affect CD47 membrane localization or inhibition of T-cell receptor (TCR) signaling by TSP-1. Notably, different molecular weights have been reported for CD47 proteins by independent studies (2, 23, 47), suggesting that the degree of N-glycosylation and glycosaminoglycan modification (or other unidentified PTMs) varies across cells. Inhibition of pGlu formation by glutaminyl cyclase isoenzyme (QPCTL), another PTM, affects the CD47–SIRPα interaction and enhances the antitumor effect (24, 25); epidermal growth factor receptor (EGFR) -induced and c-Src-mediated CD47 phosphorylation inhibits TRIM21-dependent ubiquitylation and degradation of CD47, promote tumor immune evasion (48); suggesting that PTMs play an important role in the function and clinical application of CD47.

This review discusses the different structures and functions of CD47 proteoforms, generation and function of alternatively spliced CD47 isoforms and domains, and changes in the interactions between post-translationally modified CD47 proteins and their ligands. It also explores the importance of pre-translational alternative cleavage and polyadenylation (49, 50) and clustering of the protein in aging and cancer cells, which alters the localization and affinity of CD47 for the ligand TSP-1. This review helps to understand the biology of CD47, and provides valuable new information that may help develop effective CD47-targeting therapies for the treatment of cancers, autoimmune diseases, and transplant rejection.

## 2 Characterization of CD47 protein structure

CD47 is a variably glycosylated atypical member of the immunoglobulin superfamily and an integral membrane protein consisting of an extracellular IgV-like domain, five membrane-spanning regions with short intervening loops, a short C-terminal cytoplasmic tail, and an 18-residue N-terminal signal peptide (3–5). The CD47 protein contains disulfide bonds that are conserved in all members of the family, link Cys at positions 33 to 263, connect the IgV domain to the transmembrane domain, and require positions 41 to 114 within the IgV domain (2, 51).

We analyzed the protein sequences of CD47 available from NCBI (https://www.ncbi.nlm.nih.gov/protein), and predicted theoretical molecular weight using Expasy Compute pI/Mw (https://web.expasy.org/compute_pi/), concluded that the human CD47 protein has a theoretical molecular weight of 31.88–35.22 kDa and consists of 293–323 amino acids, whereas the mouse CD47 protein has a molecular weight of 31.71–37.3 kDa and comprises 291–342 amino acids. This large variation in size is mostly due to the variable length of the C-terminal region (from being nearly absent to being more than 34 amino acids in length (5)) and the presence or absence of 21 amino acids in the extracellular domain encoded by exon 3 (which is unique to mouse CD47).

However, the molecular weights of most of the previously detected CD47 proteins were significantly greater than the theoretical molecular weights because of PTMs. For example, heterogeneous N-linked glycosylation of CD47 at asparagine residues 23, 34, 50, 73, 111, and 206 resulted in a typical diffuse migration of 40–60 kDa on SDS gel electrophoresis (2, 47, 52); heparan and chondroitin sulfate glycosaminoglycan modification at Ser64 and Ser79 generated a protein with an apparent molecular mass of >250 kDa (23). The detailed mechanisms and functions of these structural and molecular weight differences in CD47 proteins caused by alternative splicing and PTMs are discussed in the following sections.

## 3 Alternative splicing

### 3.1 CD47 isoforms generated through alternative splicing

**Figure 1:**
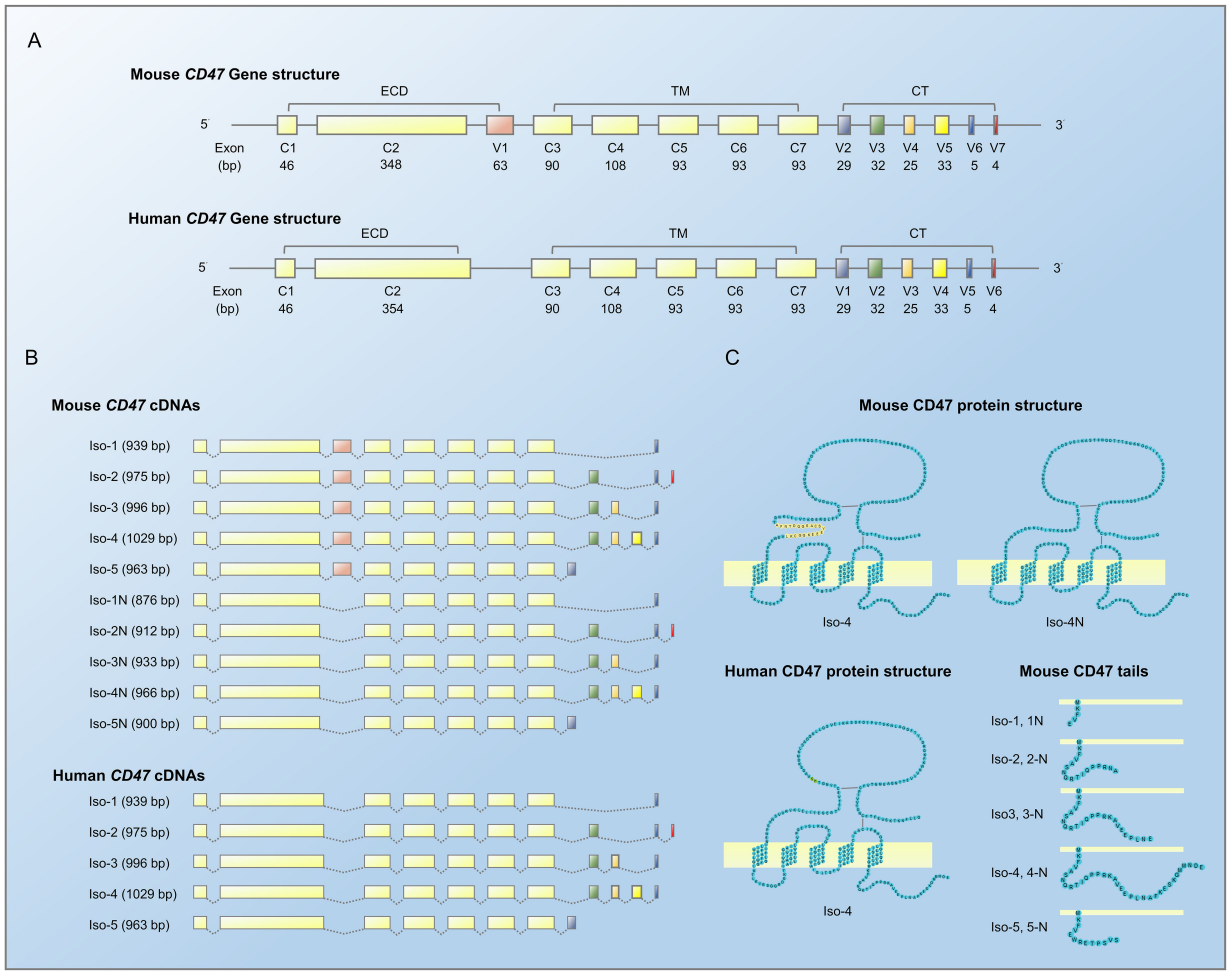
Gene and protein structures of mouse and human CD47. **(A)** Schematic diagram of an alternatively spliced CD47 gene. Light yellow denotes constant exons, encoding the extracellular domain (ECD) and multiple membrane-spanning (MMS) domain; other colors denote variant exons, encoding the cytoplasmic tail (CT) and ECD. **(B)** Several transcription products of the CD47 gene. **(C)** Structure of alternatively spliced CD47 proteins. The ECD and CT of mouse CD47 are alternatively spliced, resulting in 10 isoforms with two IgV domains (top) and five tails (bottom, right), the longest tail of which for isoforms 4 and 4-N are shown in their entirety (top, left).

Four alternatively spliced isoforms of *CD47* mRNA have been reported in various human and mouse cell lines and tissues (5). Based on the sequencing data for human and mouse CD47 (https://www.ncbi.nlm.nih.gov/genome/gdv/browser/nucleotide/?id=NM_198793.3 and https://www.ncbi.nlm.nih.gov/genome/gdv/browser/nucleotide/?id=NM_001368416.1, respectively) and a recent study (53), to date, there are five known alternatively spliced isoforms for human CD47 and 10 for mouse CD47; this difference is attributed to the fact that the mouse CD47 gene has 14 exons, whereas the human CD47 gene has 13 exons (**Figures 1A, B**; **Table 1**). The five individual human CD47 isoforms have five different tails due to the skipping of exons 8–13, whereas murine CD47 has an additional extracellular domain due to the skipping of exon 3 (**Figure 1C**). Consistent with the approach of Reinhold et al. (5), we named isoforms 1–4 based on the increasing length of the tail and isoform 5, with a completely different tail, and isoforms 1N–5N base on the 23 amino-acid deletions in the extracellular domain (**Figures 1B, C**). Isoform 5 was newly discovered by sequence analysis of cloned cDNA from human skeletal muscle; the 29-bp exon encoding the cytoplasmic tail has an amino-acid sequence completely different from that of all previously published CD47 isoforms (27). Exon 2 of human CD47 mRNA is six bases longer than that of mouse CD47, resulting in the IgV domain of human CD47 being two amino acids longer (**Figure 1C**).

**Table 1:** CD47 isoforms in different species.

Mouse CD47					Human CD47			Pig CD47		
Isoform		Total length	Exon 3	Tail length	Total length	Exon 3	Tail length	Total length	Exon 3	Tail length
1	1	312 aa	+	4 aa	293 aa	–	4 aa	–		
	1-N	291 aa	–							
2	2	324 aa	+	16 aa	305 aa	–	16 aa	303 aa	–	16 aa
	2-N	303 aa	–							
3	3	331 aa	+	23 aa	312 aa	–	23 aa	310 aa	–	23 aa
	3-N	310 aa	–							
4	4	342 aa	+	34 aa	323 aa	–	34 aa	321 aa	–	34 aa
	4-N	321 aa	–							
5	5	320 aa	+	12 aa	301 aa	–	12 aa	330 aa	–	43 aa
	5-N	299 aa	–							

aa, amino acids; +, present; -, absent.

### 3.2 Conservation of CD47 alternative splicing

Systematic analysis of human, porcine, and murine CD47 protein sequences from NCBI (https://www.ncbi.nlm.nih.gov/protein) revealed that the alternatively spliced CD47 isoforms, whether conventional isoforms 1–4 or the novel isoform 5 with a completely different tail, are highly conserved in humans and mice, but isoform 5 has not yet been reported in any other species. In addition, the deletion of 23 amino acids in the extracellular domain caused by exon 3 skipping has only been reported in mouse CD47. The alternatively spliced isoforms of mouse, human and porcine CD47 are all produced by exon skipping. Four isoforms of porcine CD47 have been identified, containing the conventional isoforms 2, 3, 4 and an isoform 5 with a 43 aa tail (distinct from human and mouse isoform 5, which have a 12 aa tails), but lacking isoform 1, which has the 4 aa tail (**Table 1**). Studies have reported on the functional differences between the conventional isoforms 1–4 (10, 12, 54–57); however, whether interspecies differences in isoform structures have functional implications requires further investigation.

### 3.3 Functions of alternatively spliced isoforms

Evolutionary conservation and tissue specificity (3) imply that functional differences occur between CD47 isoforms and that alternatively spliced isoforms of CD47 can engage in different roles under various physiological and pathological conditions. Isoform 2 is expressed in all tissues and cells, although it was primarily detected on bone marrow-derived cells and endothelia, whereas isoform 4 is highly expressed in the brain and peripheral nervous system (5, 54). Isoform 1 is expressed in keratinocytes (5) and some tumors, such as T24 bladder carcinoma (5), ovarian carcinoma cell lines (1), and breast cancer cells and biopsies (58). Isoforms 3 and 4 are thought to be closely associated with memory consolidation, based on the marked increase in their expression in rats with good memory (54, 59). However, these studies focused only on the expression of each isoform and not on their function.

Cells can adapt to some physiological and pathological changes by isoform switching. For instance, primary mouse endothelial cells cultured *in vitro* predominantly express *CD47* isoform 2 mRNA, but endothelial cells transformed with middle T antigen express CD47 isoforms 1-4. Human monocytes predominantly express isoform 2 mRNA; however, when matured into macrophages after *in vitro* culture for 7 d, all four isoforms were detected (5). In addition, alternatively spliced CD47 isoforms were upregulated in therapy-resistant pediatric acute myeloid leukemia stem cells, and a selective splicing modulator rebecsinib reversed the splicing deregulation and prevented expansion of the pediatric acute myeloid leukemia stem cells (60).

Nevertheless, other studies found no difference in the expression of CD47 isoforms. For instance, CD47 monoclonal antibody induced similar levels of apoptosis in JinB8 cells, which express human CD47 isoforms 1 and 2 equally (55); forced expression of mouse CD47 isoform 2 or 4 in N1E-115 neuroblastoma cells induced marked neurite and filopodium formation (10); all four isoforms interacted with αv integrin to equal degrees to mediate vitronectin(Vn)-bead binding (56); and isoforms 1 and 2 activated T cells in both humans and mice to equal degrees (12, 57). These findings suggest that CD47 isoforms play indistinguishable roles. Nevertheless, CD47 isoform functions and switching during cellular transformation, tumorigenesis and tumor development, and neurotransmission require further investigation.

### 3.4 Functions of cytoplasmic tails

PLIC-1 and BNIP3 have been identified as direct cytoplasmic binding partners. The cytoplasmic protein PLIC-1 binds directly to the C-terminal cytoplasmic tail of CD47 isoforms 2 and 4, thereby promoting cell spreading, altering intermediate filament distribution (13), recruiting heterotrimeric G proteins to CD47 (61, 62), inhibiting chemotaxis signaled by the Gi-coupled receptor CXCR4 (62), activating the PI3K/Akt pathway in astrocytoma cells (63), and regulating cAMP signaling by CD47. BNIP3 has been identified as another possible cytoplasmic binding partner via yeast two-hybrid screening using the 145-amino-acid C-terminal (corresponding to the multi-transmembrane and cytoplasmic tail) of CD47 isoform 2 as bait. Under basal conditions in Jurkat leukemic T cells, CD47 and BNIP3 colocalize on the membrane after T-cell stimulation via CD47 by the TSP-1-derived peptide 4N1K, resulting in the translocation of BNIP3 to the mitochondria to induce mitochondrial depolarization and Jurkat leukemic T cells death. However, binding to CD47 requires the transmembrane domain of BNIP3, suggesting a partially lateral interaction (14).

Furthermore, structure–function studies in cell lines transfected with various CD47 chimeras and plated on SIRPα–Fc demonstrated that the C-terminal cytoplasmic tail was partially effective in CD47-dependent filopodium formation. This was mediated predominantly through the activation of Cdc42, as the effect of the mutant lacking the cytoplasmic tail was slightly weaker than that of wild-type CD47 (10). However, the cytoplasmic tail is dispensable in many processes such as neurite formation by forced CD47 expression (10), apoptosis of normal and leukemic cells (55), neurite and filopodium formation (64), cell adhesion and lamellipodium formation (65), CD47 localization of hippocampal neurons (66), Vn-bead binding (56), T-cell activation (12, 57), and platelet aggregation (67). Although most studies have shown that the tail of CD47 is dispensable, these studies may be limited by the fact that the cells they used expressed different isoforms with different tails; thus, the function of the cytoplasmic tail requires further investigation.

### 3.5 Functions of the IgV and MMS domains

Many functions of the IgV and MMS domains in CD47 signaling have been elucidated in studies using single-domain mutants, as described below. Some important functions of CD47 have been attributed to the IgV domain alone, which is responsible for the interaction between CD47 and integrins. In the human B-lymphocytic cell line, Namalwa cells, and Chinese hamster ovary (CHO) cells, the truncated form of CD47 with only the IgV domain (abbreviated to CD47 IgV domain) was sufficient for activating αIIbβ3 without intracellular signaling when binding to TSP-1 (67). In the ovarian carcinoma cell line OV10, Vn-bead binding was CD47- and integrin-dependent and correlated with both integrin αv and CD47 expression, and the CD47 IgV domain alone was sufficient (56). Another study showed that CD47 complexed via its IgV domain, with αvβ3 in cyclodextrin-resistant domains promoted integrin activation induced by RGD peptide and αvβ3 avidity for binding immobilized substrates by enhancing integrin clustering. These CD47–αvβ3 complexes differed from those localized in cholesterol-rich domains, which appeared to be involved in Gi-dependent signaling, required both the MMS and IgV domains, and were disrupted by cyclodextrin treatment (68). CD47 promoted neurite formation in mouse neuroblastoma N1E-115 cells by activating the members of the Rho family of small G proteins Rac and Cdc42; here, the CD47 IgV domain was sufficient, and the MMS domain and short cytoplasmic tail were dispensable. Although the CD47-dependent formation of filopodia in cells plated on SIRPα–Fc appeared to be mediated predominantly through the activation of only Cdc42, the entire structure of CD47 was required for maximal enhancement of filopodium formation (10).

The coexistence of the IgV and MMS domains is required for the co-stimulation of T cells and TCRs in both humans and mice via an adhesion-dependent and CD28-independent signaling pathway (12). This enhances the efficiency of TCR signaling by causing T cells to spread on an antigen-presenting cell or surface (57) and promote caspase-independent B-CLL cell death through CD47-stimulated phagocytotic signaling by human dendritic cells (55). CD47 participates in the constitutive arrest of T cells on inflamed vascular endothelium by upregulating the expression of α4β1 integrins, where the IgV domain, the first transmembrane domain, and a short intracytoplasmic loop of 12 amino acids are sufficient (69). CD47 also participates in the regulation of cell–cell adhesion and cell migration through the reorganization of the actin cytoskeleton in epithelial cells, as mediated by the activation of Src and mitogen-activated protein kinase. The MMS domain is required for the localization of CD47 at cell–cell adhesion sites and lamellipodium formation, whereas the IgV domain participates in lamellipodium formation (65). The MMS domain is involved in maintaining an appropriate CD47 topology (51, 70, 71). The findings of these functional studies are summarized in **Table 2**.

**Table 2:** Functions of CD47 isoforms and domains.

Functions	Cell type	IgV domain	MMS domain	Cytoplasmic tails	Isoforms
CD47 interacts with αv integrin to mediate Vn-bead binding (56).	Human ovarian carcinoma cell line OV10	Sufficient	Dispensable	Dispensable	Four isoforms contribute equally.
CD47–αvβ3 complexes in cyclodextrin-resistant domains promote integrin activation induced by RGD peptide and αvβ3 avidity for binding immobilized substrates by enhancing integrin clustering (68).		Sufficient	Dispensable	–	–
CD47–αvβ3 complexes in cyclodextrin-sensitive cholesterol-rich raft are involved in Gi-dependent signaling (68).		Necessary	Necessary	–	–
PLICs bind to cytoplasmic tails of CD47 and induce the redistribution of vimentin and cell spreading (13).		–	–	Tails of iso-2 and iso-4 directly interact with PLICs.	
CD47 costimulates T-cell activation with TCR via an adhesion-dependent and CD28-independent signaling pathway (12).	Human T lymphocyte leukemia cell line Jurkat	Necessary	Necessary	Dispensable	Iso-1 and iso-2 contribute equally.
CD47 costimulates T-cell activation and enhances TCR signaling by promoting T-cell spread on APC or surface (57).		Necessary	Necessary	Dispensable	Iso-1 and iso-2 contribute equally.
The long-range disulfide bond between the IgV and MMS domains is required for binding of anti-Ig domain monoclonal antibodies and SIRPα (51).		Necessary	Necessary	–	–
CD47 induces caspase-independent cell death (normal and leukemic cells) by triggering phagocytotic signaling by human DCs (55).		Necessary	Necessary	Dispensable	Iso-1 and iso-2 contribute equally.
BNIP3 colocalizes with CD47 on the membrane but translocates to the mitochondria to induce mitochondrial depolarization and T-cell death after stimulation via CD47/TSP-1 (14).		Ineffective	Necessary	Inconclusive	–
CD47 interacts with and switches αIIbβ3 to a high-affinity state to induce platelet aggregation (67).	Human B-lymphocytic cell line Namalwa; Chinese hamster ovary (CHO) cells	Sufficient	Dispensable	Dispensable	–
CD47 promotes neurite formation by activating the members of the Rho family of small G proteins Rac and Cdc42 (10).	Mouse neuroblastoma cell line N1E-115; H-Ra-transformed CHO cell	Sufficient	Dispensable	Dispensable	Iso-1 and iso-2 contribute equally.
CD47-dependent formation of filopodia in cells plated on SIRPα–Fc is mediated predominantly through Cdc42 activation (10).		Partially effective	Partially effective	Partially effective	–
Differential localization of SIRPα and CD47 at axons and dendrites regulates synaptogenesis and formation of neural networks (66).	Hippocampal cells	Necessary	Ineffective	Dispensable	–
CD47 is thought to be closely associated with memory consolidation in rats (54).		–	–	–	Iso-3 and iso-4 are associated with memory consolidation.
CD47 regulates cell–cell adhesion and cell migration through reorganization of actin cytoskeleton in epithelial cells (65).	Madin–Darby canine kidney cells	Partially participates in lamellipodium formation.	Required for localization of CD47 at cell–cell adhesion sites and lamellipodium formation.	Dispensable	–

IgV, immunoglobulin variable; Iso, isoform; MMS, multiple membrane-spanning; TCR, T-cell receptor; -, no data.

## 4 PTMs

### 4.1 Disulfide bond

The formation of disulfide bonds is essential for the function and stability of various proteins, including CD47. Both the IgV and MMS domains are required to mediate virtually all the functions of CD47 and for its efficient localization to membrane rafts, indicating that the interaction between these domains may facilitate protein function and subcellular localization. The disulfide bonds of the CD47 protein are conserved in all members of the family, linking Cys at positions 33 to 263, the IgV domain to the MMS domain, and positions 41 and 114, which are necessary for IgV domain formation (2, 4, 51). Subtle changes in CD47 conformation, in the absence of the long-range disulfide bond between Cys33 and Cys263, are indicated by decreased binding between two anti-Ig domain monoclonal antibodies, decreased SIRPα binding, and reduced CD47/SIRPα-mediated cell adhesion. Mutagenic disruption of this disulfide bond completely inhibited CD47 signaling independent of ligand binding, as indicated by T cell-mediated interleukin-2 secretion and Ca^2+^ responses. Loss of the disulfide bond did not affect the membrane raft localization of CD47 or its association with αvβ3 integrin (51). Other structural studies indicated that the MMS domain is crucial for establishing the disulfide bond in CD47 and maintaining an appropriate topological structure (70, 71).

### 4.2 Glycosylation and glycosaminoglycan

CD47 is a heavily glycosylated protein with six potential N-linked glycosylation sites at asparagine residues 23, 34, 50, 73, 111, and 206 (sequences containing 18 signal peptides), five of which are in the IgV domain. Sequencing studies of erythrocytes indicated that residues 34, 73, and 111 of CD47 are glycosylated, residues 23 and 50 are unknown, and residue 206 at the C-terminus is not glycosylated (2). Glycosylation may be expected at Asn23 and Asn50 because N-glycosylation occurs preferentially at sites near the N-terminus of proteins (72). Glycosylation is thought to influence the localization of CD47 on the cell membrane. Site-by-site mutagenesis of five N-glycosylation sites progressively downregulated yeast CD47 expression, indicating that glycosylation regulates heterologous display on yeasts (20). However, in Sf9 insect cells, core glycosylation was sufficient for exporting at least the soluble form of human CD47 (21). Similarly, in CHO cells, removal of all N-linked sugars promoted the export of mouse but not of human CD47, and human CD47, with all five of the N-linked glycosylation sites knocked out, could still bind to soluble SIRPα, indicating that N-glycosylation sites are not required for SIRPα binding (22).

Glycosaminoglycans interact with various proteins involved in development and homeostasis, with heparan sulfate and/or chondroitin/dermatan sulfate as the more common physiological ligands (73). The CD47 proteoglycan undergoes glycosaminoglycan modification at Ser64 and Ser79 (sequences excluding 18 signal peptides) by heparan and chondroitin sulfate. This proteoglycan isoform of CD47 is widely expressed on vascular cells. Mutagenic studies have identified glycosaminoglycan modification of CD47 at Ser64 and Ser79 and that the inhibition of TCR signaling by TSP-1 is mediated by CD47, requiring its modification at Ser64 (23).

### 4.3 Ubiquitination and pyroglutamation

Few studies have investigated CD47 ubiquitination. A bioinformatics study (data from http://ubibrowser.ncpsb.org/) predicted seven E3 ligases for human CD47, six for mouse CD47, and two for pig CD47, with a high confidence level (74). Furthermore, human CD47 isoforms 1, 2, and 3 have possible ubiquitination sites at K74 (not conserved in mouse CD47), and human isoform 4, which has the longest tail, has two additional sites in its tail at K314 and K317 (conserved in mouse CD47; data from https://gygi.med.harvard.edu/ggbase/). Nevertheless, further research is required to verify these modifications and their functions. A recent study identified two ubiquitylation sites on CD47 that are distinct from the putative sites mentioned above and showed that in human glioma cells, activated EGFR enhanced Src-mediated CD47 Y288 phosphorylation, which blocked binding of the E3 ubiquitin ligase TRIM21 to CD47 and prevented TRIM21-mediated CD47 K99/102 polyubiquitylation and CD47 degradation, resulting in increased CD47 protein levels, which led to inhibition of phagocytosis by tumor-infiltrating macrophages and ultimately facilitated tumor immune escape (48).

The N-terminal pGlu modification of CD47 by glutaminyl-peptide cyclotransferase-like protein (QPCTL, also known as isoQC) was recently discovered to be a critical regulator of the CD47–SIRPα axis. Biochemical analysis demonstrated that QPCTL is critical for pGlu formation on CD47 at the SIRPα-binding site shortly after biosynthesis. Inhibition of QPCTL with the glutaminyl cyclase inhibitors SEN177 and PQ912 reduced the affinity of CD47 for anti-CD47 antibodies or human SIRPα fusion proteins. SEN177 suppressed the function of QPCTL and increased antibody-dependent cellular phagocytosis and cytotoxicity of anti-CD20 antibodies *in vitro*. In addition, QPCTL inhibitors, combined with anti-CD47 antibodies, enhanced the antitumor effect. Neutrophil-mediated killing of cancer cells was also substantially increased *in vivo* after treatment with QPCTL (24). Similarly, another study revealed that pyroglutamate formation catalyzed by isoQC at the N-terminus is required for CD47–SIRPα binding, suggesting that isoQC is an essential regulator of the CD47–SIRPα axis and is required for efficient phagocytic cell-mediated clearance of cancer cells. Notably, the N-terminal Q19 of CD47 was modified to a pGlu residue by isoQC (25), which is consistent with earlier findings showing that the N-terminus of CD47 is glutamate (2), which can self-cyclize to form pGlu (3). These studies also propose that the commercial monoclonal antibody CC2C6 is a pGlu-dependent antibody for CD47 (24, 25).

### 4.4 Truncated CD47 from proteolytic cleavage or exosome shedding

CD47 can be released from the cell surface by proteolytic cleavage or exosome shedding. In vascular smooth muscle cells cultured in low-glucose concentrations, proteolytic cleavage of the IgV domain is mediated by matrix metalloprotease-2 (MMP-2), and higher glucose concentrations increase CD47 stability by decreasing the levels of active MMP-2 released into the conditioned medium. The proteolytically cleaved CD47 may bind to TSP-1 and SIRPα (26), although the signaling function for this fragment remains unclear.

CD47 bearing the glycosaminoglycan modification required for TSP-1 signaling has been detected in culture supernatants of endothelial, vascular smooth muscle, CHO, and T cells (23), suggesting that CD47 in extracellular vesicles may participate in extracellular signaling. CD47 has also been detected in exosomes released by platelets (27), erythrocyte-derived microvesicles (28), human mesenchymal stem cells (30), T cells (31), and CD47-overexpressing normal or tumor cells (transgenic or native) (32). TSP-1 and its receptor CD47 are expressed on T cell-derived exosomes/ectosomes, which are internalized, regulate the activation of T cells by engaging the TCR, and are taken up by recipient endothelial cells, thereby altering the global gene expression to modulate endothelial cell responses to vascular endothelial growth factor and tube formation in a CD47-dependent manner (31). Extracellular vesicles, including exosomes, released from CD47-overexpressing normal or tumor cells (transgenic or native), can induce efficient CD47 cross-dressing on pig or human cells, which can interact with SIRPα to inhibit phagocytosis without transmitting harmful cellular signals (32).

## 5 Alternative cleavage and polyadenylation generate 3′ untranslated regions of isoforms

Approximately half of the human genes undergo alternative cleavage and polyadenylation to generate mRNA transcripts that differ in the length of their 3′ untranslated regions (UTRs) but produce the same protein. Many previous functional studies have used the 3’ UTR-free CD47, but it was reported that CD47 mRNA 3’ UTRs are involved in regulation of CD47 cell surface expression: the long 3′ UTR of CD47 mRNA promotes cell surface expression of the protein, whereas the short 3′ UTR promotes the cytoplasmic retention of the protein in the endoplasmic reticulum (49). In the same model, the long 3′ UTR of CD47 mRNAs acts as scaffolds that recruit protein complexes promoting the interaction between SET and newly translated CD47 cytoplasmic domains, resulting in the translocation of CD47 to the plasma membrane (49, 50). A recent study revealed that this mechanism of modulation of CD47 expression has a role in muscle regeneration (75) and reported that elevated CD47 levels on aged MuSCs with impaired regenerative capacity resulted from increased U1 snRNA expression, which disrupts alternative polyadenylation to generate a long 3’ UTR of CD47 mRNA. Antibody blockade of TSP-1/CD47 signaling reversed sarcopenia and restored muscle mass and function in aged mice, suggesting therapeutic implications (75).

## 6 Clustered distribution of CD47

Apoptotic cells show altered spatial distributions (76–78) of CD47 expression that can render the CD47–SIRPα inhibitory signal ineffective. CD47 molecules on T lymphocyte leukemia cell line Jurkat are evenly distributed under normal conditions but are clustered during apoptosis, leaving calreticulin and phosphatidylserine exposed to trigger phagocytosis (77). In contrast, CD47 distribution on human colorectal adenocarcinoma cells changes from a clustered to diffuse pattern during apoptosis, which is associated with reduced binding to SIRPα, leading to increased phagocytosis (78). CD47 on experimentally aged red blood cells (RBCs) undergoes a conformational change upon binding to TSP-1 or TSP-1-derived peptide 4N1K that alters CD47 expression; binding to TSP-1 generates a pro-phagocytic signal (76). Coincidently, a conformational change in CD47, as identified by anti-CD47 monoclonal antibody 2D3 on sickle RBCs, is also associated with an increase in TSP-1-binding capacity (79).

Because the diffraction-limited resolution of conventional fluorescence microscopy and flow cytometry of 2D3-antibody binding do not allow for visualization of conformational changes, direct stochastic optical reconstruction microscopy-based imaging, coupled with quantitative analysis, provides a more suitable approach for exploring structural changes in CD47-nanoscale protein clusters. This approach provides clear nanoscale images of aging-associated changes in CD47 distribution. CD47 molecules in young RBCs reside as nanoclusters with limited binding to TSP-1, whereas those in aged RBCs decrease in number and form bigger and denser clusters with increased TSP-1-binding ability. Exposure of aged RBCs to TSP-1 further increased the size of CD47 clusters via a lipid raft-dependent mechanism (80).

## 7 Conclusion and future perspectives

CD47 is a multifunctional protein that mediates several cellular processes, including apoptosis, proliferation, adhesion, and migration, upon engagement with its ligands. It is widely recognized for its role in the immune system, acting as a “don’t-eat-me” signal to protect cells from phagocytosis upon interaction with SIRPα on macrophages, dendritic cells, and natural killer cells. Emerging evidence demonstrates that CD47 plays an important role in cancer immunotherapy and transplant rejection: 1) elevated expression of CD47 was observed in a wide range of cancer cells, acting as a mechanism for evading the immune system (33, 34, 36); 2) hematopoietic stem/progenitor cells upregulated their CD47 expression upon mobilization for protection against phagocytosis by circulating macrophages (35); and 3) CD47 overexpression inhibited immune rejection of allogeneic and xenogeneic grafts (41, 81, 82). Thus, CD47 blockade is considered an effective approach to improve cancer immunotherapy; transgenic overexpression or regulation of CD47 is expected to ameliorate the development and symptoms of autoimmune diseases and transplant rejection and enable the generation of hypoimmunogenic pluripotent stem cells.

**Figure 2:**
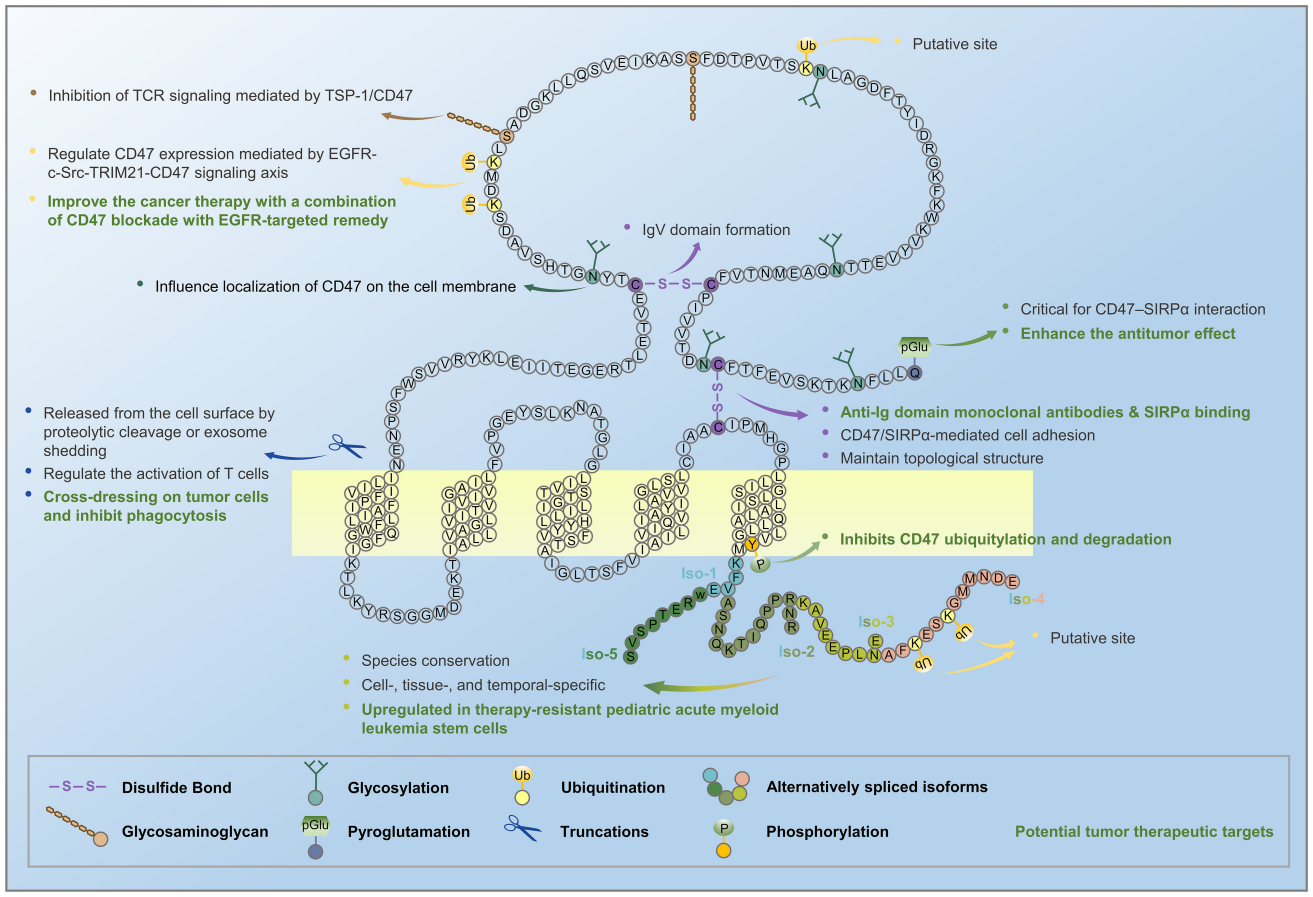
Alternative splicing and post-translational modifications of CD47 generate several CD47 proteoforms. CD47 mRNA undergoes alternative splicing to form five isoforms, and CD47 protein undergoes post-translational modifications, such as glycosylation, glycosaminoglycan, pyroglutamate, proteolytic cleavage, and exosome shedding.

For a long time after the discovery and characterization of CD47, apart from a large number of studies on CD47 proteoforms (produced from a single gene by alternative splicing and other PTMs) during the first decade, there were not many new findings, leading to a gradual neglect of this research topic. However, with the discovery of important functions of the CD47 proteoform in recent years, we need to revisit this issue. Functional differences between CD47 isoforms and the regulation of alternatively spliced isoforms represent a potentially clinically translatable strategy for tumor therapy, given that the upregulated expression of alternatively spliced CD47 isoforms in therapy-resistant pediatric acute myeloid leukemia stem cells can be reversed by the selective splicing modulator rebecsinib (60, 83). In addition to the traditional methods of blocking CD47 with antibodies or SIRPα or genetically downregulating CD47 expression to enhance antitumor immune responses, QPCTL inhibitors, combined with anti-CD47 antibodies, can enhance the antitumor effect by blocking the post-translational pGlu modification catalyzed by QPCTL, which is critical for CD47–SIRPα interaction (24, 25). In EGFR-activated tumor cells, CD47 phosphorylation inhibits ubiquitylation and degradation of CD47, leading to upregulation of CD47, indicating the role of these PTMs in tumor evasion and the potential to improve the current cancer therapy (48) (**Figure 2**). Elevated CD47 levels on aged MuSCs with impaired regenerative capacity resulted from increased U1 snRNA expression, which disrupted alternative polyadenylation, generating a long 3’-UTR of CD47 mRNA (75). CD47 released from CD47-overexpressing normal or tumor cells (transgenic or native) could induce efficient CD47 cross-dressing on pig or human cells, which could interact with SIRPα to inhibit phagocytosis, and may also be used as a new tumor therapy approach in the future (32).

The proteoform of CD47 is complex, similar to its myriad of functions; thus, further investigation is needed to fully characterize the roles of CD47 in cell biology and examine how these roles are affected by alternative splicing and PTMs as well as the related changes in their expression and distribution. For example, studying the functions of alternative splicing isoforms and different domains in new models, exploring the regulatory mechanisms and roles of alternative splicing, identifying and validating new post-translational modification sites such as ubiquitination and their functions are future research directions. These studies can provide new insights on the development of therapeutic approaches in muscle regeneration, tumor therapy, autoimmune diseases, and transplant rejection.

## Author contributions

TZ: Data curation, Writing – original draft, Writing – review & editing. FW: Writing – original draft. LX: Writing – original draft. YY: Writing – review & editing.
